# Dietary, circulating beta-carotene and risk of all-cause mortality: a meta-analysis from prospective studies

**DOI:** 10.1038/srep26983

**Published:** 2016-05-31

**Authors:** Long-Gang Zhao, Qing-Li Zhang, Jia-Li Zheng, Hong-Lan Li, Wei Zhang, Wei-Guo Tang, Yong-Bing Xiang

**Affiliations:** 1 Shanghai Cancer Institute, Renji Hospital, Shanghai Jiaotong University School of Medicine, Shanghai, China; 2 University of South Carolina, Arnold School of Public Health, Department of Epidemiology and Biostatistics, Columbia, SC29208, USA

## Abstract

Observational studies evaluating the relation between dietary or circulating level of beta-carotene and risk of total mortality yielded inconsistent results. We conducted a comprehensive search on publications of PubMed and EMBASE up to 31 March 2016. Random effect models were used to combine the results. Potential publication bias was assessed using Egger’s and Begg’s test. Seven studies that evaluated dietary beta-carotene intake in relation to overall mortality, indicated that a higher intake of beta-carotene was related to a significant lower risk of all-cause mortality (RR for highest vs. lowest group = 0.83, 95%CI: 0.78–0.88) with no evidence of heterogeneity between studies (*I*^*2*^ = 1.0%, *P* = 0.416). A random-effect analysis comprising seven studies showed high beta-carotene level in serum or plasma was associated with a significant lower risk of all-cause mortality (RR for highest vs. lowest group = 0.69, 95%CI: 0.59–0.80) with low heterogeneity (*I*^*2*^ = 37.1%, *P* = 0.145). No evidence of publication bias was detected by Begg’s and Egger’s regression tests. In conclusion, dietary or circulating beta-carotene was inversely associated with risk of all-cause mortality. More studies should be conducted to clarify the dose-response relationship between beta-carotene and all-cause mortality.

It is widely hypothesized that the beta-carotene rich in green leafy vegetables and other orange-colored plant may prevent oxidative damage^1^,^2^. In addition, it is an important pro-vitamin A carotenoid that is metabolized into bioactive vitamin A by the human body^3^. Therefore, based upon the antioxidant and pro-vitamin A functions of beta-carotene, it is biologically plausible to extend the human life span.

*In vitro* animal studies have demonstrated that beta-carotene may prevent oxidative damage by counteracting the effects of free radicals^4^, which is thought to be involved in the pathological process of many chronic diseases. While high dietary intake of beta-carotene has been associated with lower risk of all-cause mortality in observational studies^5^, the results varied in other studies^6^,^7^. Besides, studies assessed the relation between circulating level of beta-carotene and risk of all-cause mortality yielded inconsistent results^8^,^9^,^10^. Additionally, clinical trials of supplementation with beta-carotene have shown no benefits and discouraging results^11^. One explanation is that different sources of beta-carotene may generate different influence in its metabolism^12^. For example, beta-carotene from natural food and supplements may have different influence on human health.

Due to inconclusive data on the effect of dietary beta-carotene intake or circulating beta-carotene levels on risk of all-cause mortality in general healthy population, it is critically important to clarify these associations in general population. Therefore, to evaluate these associations and summarize available observational evidence, we attempted to conduct a meta-analysis of prospective studies on this topic.

## Results

### Characteristics of included studies

**Figure 1:**
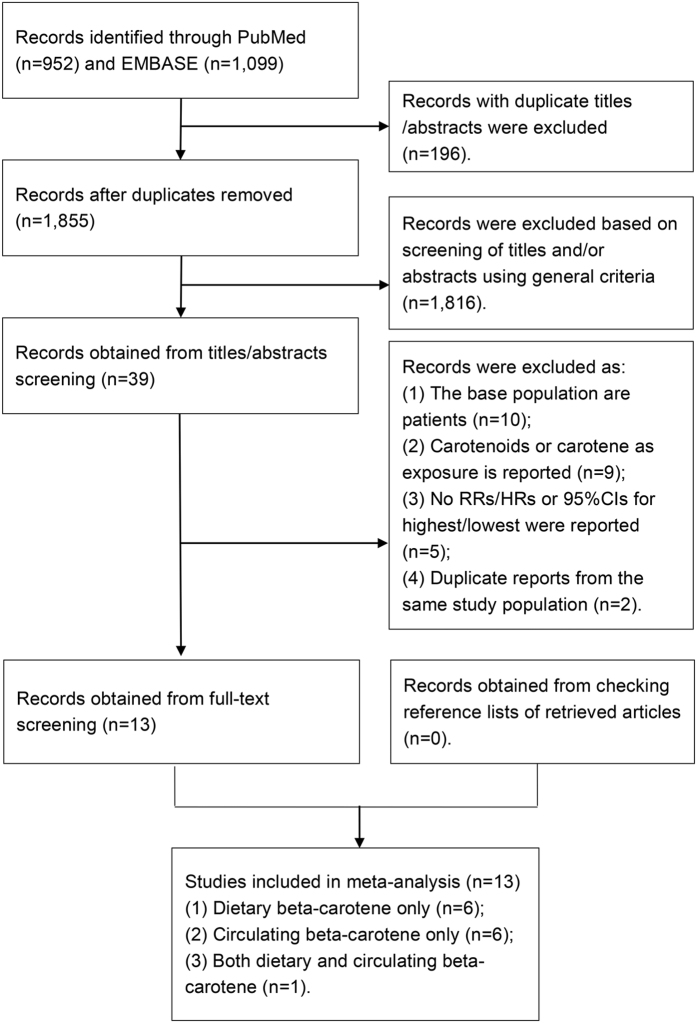
Flow diagram for selection of studies in meta-analysis of beta-carotene and risk of all-cause mortality. RR, relative risk; HR, hazard ratio; CI, confidence interval.

Figure 1 shows a flow diagram of search process and results of included studies. We identified 1,855 potentially relevant articles after removal of duplication from our preliminary search of the two databases. Of these, 1,816 were excluded according the inclusion criteria described in the methods section after a review of abstract or title, such as review, animal research and retrospective study, leaving 39 articles for a further full-text review. Among the remaining articles, ten were excluded because they were not conducted in general healthy population. These excluded studies were based on population with specific exposure or health condition, such as certain cancers, patients with obstructive lung function and asbestos-exposed workers. Additionally, nine were removed for taking total carotenoids or carotene as exposure. Two studies^9^,^13^ were not considered for duplicate reports from the same study population. Five additional studies^14^,^15^,^16^,^17^,^18^ were excluded because we cannot get sufficient data to recalculate the RR or 95%CI for the highest versus lowest group. After exclusion, 13 articles (dietary intake: seven publications; circulating concentration: seven publications)^5^,^6^,^7^,^8^,^10^,^19^,^20^,^21^,^22^,^23^,^24^,^25^,^26^ were included in this meta-analysis, of which one study^21^ reported results for both dietary and circulating level of beta-carotene, respectively.

Table 1 showed the characteristics of the 13 included articles. All of these articles were published between 1997 and 2016, consisted of 17,657 deaths among 174,067 participants. The duration of follow-up ranged from two to 25.7 years. Of the included studies, nine were conducted in Europe, two in United States, and two in Japan. Study participants in five studies are only restricted to subjects over 50 years old. All of the studies but one (included only men) included both men and women. All of the studies taking the dietary intake of beta-carotene as an interest exposure used a structured food frequency questionnaires (FFQ), among which the FFQ in four studies had been validated. Only one study^5^ included beta-carotene from supplements when calculated total beta-carotene consumption. In the seven cohorts focusing the beta-carotene concentration in blood, five tested in serum and two conducted in plasma. The included studies provided the relative risk estimates adjusted for age (13 studies), body mass index (9 studies), smoking status (8 studies), alcohol consumption (8 studies), energy intake (6 studies), physical activity (7 studies), and history of chronic diseases (6 studies).

### Dietary intake of beta-carotene and all-cause mortality

**Figure 2:**
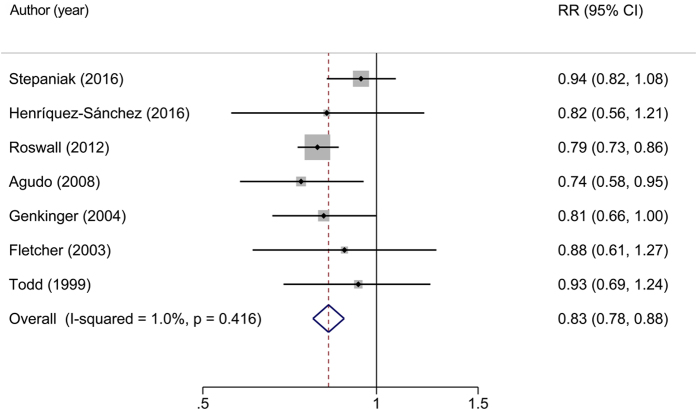
Relative risks of all-cause mortality for highest versus lowest category of dietary intake of beta-carotene. Overall relative risk calculated with random effects model.

Seven studies^5^,^6^,^7^,^19^,^20^,^21^,^22^ including 11,810 deaths among 149,774 cohort members were included in the analysis, which indicated that a high intake of beta-carotene was related to a significant reduced risk of all-cause mortality (RR for highest vs. lowest group = 0.83, 95%CI: 0.78–0.88) with no evidence of between study heterogeneity (*I*^*2*^ = 1.0%, *P* = 0.416) under a random-effect model (Fig. 2).

In subgroup analyses, the associations between dietary intake of beta-carotene and risk of all-cause mortality did not differ substantially by number of participants, age at baseline, measurement type of exposure, median intake and adjustment of energy intake, vitamin supplements use and serum cholesterol level. The exception is that an inverse association was not significant in studies failed to adjust chronic disease history (Table 2).

To explore the influence of multi-variables adjustment, several sensitivity analyses were carried out. We conducted the analyses by excluding a study^7^ that did not adjust for energy intake and a study^6^ that did not provide information about median or mean intake. Overall, the sensitivity analyses did not lead to any changes in the significance or direction of effect for the association between dietary intake of beta-carotene and risk of all-cause mortality. Moreover, we removed one study at a time sequentially to reanalysis the data with the risk estimates ranging from 0.80(95%CI: 0.75–0.86) when excluding the study by Stepaniak^19^, to 0.87(95%CI: 0.80–0.96) after omission of the study by Roswall^7^. None of the studies considerably affected the summary results.

### Circulating level of beta-carotene and all-cause mortality

**Figure 3:**
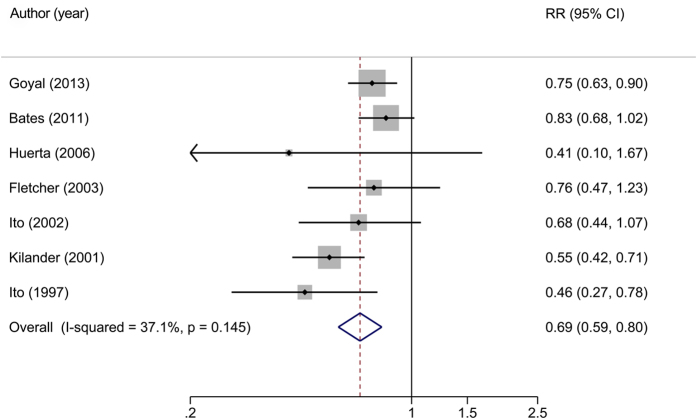
Relative risks of all-cause mortality for highest versus lowest category of circulating level of beta-carotene. Overall relative risk calculated with random effects model.

The relation between circulating concentration of beta-carotene and risk of all-cause mortality was evaluated in seven studies^8^,^10^,^21^,^23^,^24^,^25^,^26^ comprised 25,468 participants with 6,137 deaths. A random-effect analysis showed a high beta-carotene level in serum or plasma was associated with a significant reduced risk of all-cause mortality (RR for highest vs. lowest group = 0.69, 95%CI: 0.59–0.80) with low heterogeneity (*I*^*2*^ = 37.1%, *P* = 0.145) (Fig. 3).

Subgroup analyses were performed across a number of key study characteristics (Table 3). The significance of stratified pooled RRs slightly differed when classified by whether adjusting for history of chronic diseases, BMI, physical activity, vitamin supplements use and serum cholesterol level. Stratifications by other characteristics did not have a substantial impact on the major results. In general, results from the meta-regression analysis indicated that no significant heterogeneity was observed between subgroups.

To further confirm the robustness of the results, we conducted sensitivity analyses by excluding sequentially one study at a time. The pooled risks ranged from a low estimate of 0.67(95%CI: 0.59–0.76) when removing the study by Bates^10^, to a high estimate of 0.72(95%CI: 0.65–0.81) while omitting the study by Ito^25^ but were similar in general. In main analyses, we transformed RRs for per standard deviation (SD) into RRs for top versus bottom tertile in two studies^10^,^26^. After excluded these two studies, the risk estimates did not change much (RR = 0.71, 95%CI: 0.61–0.82, *I*^*2*^ = 0.0%, *P* = 0.458).

### Publication bias

In analysis of dietary intake of beta-carotene and all-cause mortality, visual inspection of Begg’s and Egger’s regression tests provided no evidence of publication bias (Begg’s test: *P* = 0.764; Egger’s test: *P* = 0.567). Similarly, no publication bias was observed by the funnel plot, Egger’s regression test (*P* = 0.209), or by Begg’s rank correlation test (*P* = 0.368) in the meta-analysis on association between circulating concentration of beta-carotene and risk of all-cause mortality. Funnel plots are provided in supplemental materials (Supplemental Figs 1 and 2).

## Discussion

In the present meta-analysis, the dietary intake of beta-carotene was inversely associated with risk of all-cause mortality in general population. The association was consistent in subgroup and sensitivity analyses. In addition, the high level of circulating beta-carotene was also associated with a lower risk of all-cause mortality. No publication bias was detected in our analyses.

In contrast with results from randomized control trials, we observed a favorable impact of dietary or circulating beta-carotene on risk of all-cause mortality among general population in observational studies. A review^11^ combining 26 trials reported that the high-dose supplementation of beta-carotene may lead to null or adverse effect on all-cause mortality. The discrepancy between intervention trials and cohort studies may be explained by several reasons. Firstly, beta-carotene in natural or synthetic forms may have difference in bioavailability on risk of all-cause mortality. Natural source of beta-carotene may be due to synergistic interaction with other micronutrients present in non-processed or natural food^27^,^28^. Secondly, another explanation is that the dose of beta-carotene supplement used in intervention studies is higher than that in most epidemiological studies. A meta-analysis of randomized control trials indicated that beta-carotene supplements used separately or together with other antioxidants significantly increased mortality in population with doses above the recommended daily allowances. In contrast, non-significantly inverse association was observed in groups below recommended daily allowances^29^. The underlying mechanism may be the possibility of a U-shaped relation between beta-carotene status and mortality risk^8^,^30^,^31^. From this point of view, in poorly nourished populations, a higher intake of beta-carotene may decrease the risk of all-cause mortality^32^. While in population with a relatively high nutritional status, the benefits may disappear with additional non-dietary intake. Thirdly, the association we observed may reflect residual confounding from other important dietary ingredients, which is highly related with dietary beta-carotene. However, this cannot be ruled out based on observational studies.

Extensive evidence has demonstrated that dietary beta-carotene had protective effects in preventing non-communicable chronic disease. Recently, results from 37,846 participants of the European Prospective Investigation into Cancer and Nutrition-Netherlands study indicated that higher dietary intakes of beta-carotene were associated with a reduced diabetes risk^33^. Additionally, a systematic review demonstrated that evidence from cohort studies supported the protective effects of dietary beta-carotene on preventing cardiovascular disease^34^. In regard to cancers, some studies suggested that higher intake of dietary beta-carotene could reduce lung^35^ and colorectal cancer risk^36^. In a recent Japanese cohort study, high serum carotenoids especially alpha- and beta-carotene and beta-cryptoxanthin were associated with lower risk for the metabolic syndrome^37^. Therefore, the evidence was consistent with our results dietary or circulating beta-carotene is associated with risk of all-cause mortality.

Biologically, dietary source of beta-carotene may reduce risk of mortality in humans through several mechanisms. Firstly, beta-carotene can play an important role for its pro-vitamin A activity^2^. It is widely assumed that vitamin A is essential to human body for normal organogenesis, tissue differentiation, immune competence, and maintaining a normal vision^38^. Actually, based on current evidence, it is obvious that some under nutritional population do not meet the recommendation for vitamin A intake^3^. Secondly, beta-carotene may exert physiological action by both antioxidant and prooxidant effects. The antioxidant-prooxidant activity of beta-carotene would be dependent on the oxygen tension in human body^39^. With a low oxygen tension, beta-carotene and other antioxidants can act synergistically as an effective radical-scavenging antioxidant in biological membranes^40^. The antioxidant properties of beta-carotene seemed to protect against chronic diseases and conditions, such as heart disease, stroke, cancer, diabetes and obesity^41^,^42^. Thirdly, beta-carotene may enhance immune cell function to play a major role in the prevention of chronic diseases^43^.

Strengths of our studies included the large number of both total participants and outcomes. Additionally, the results were stable and robust in subgroup and sensitivity analyses. In current analysis, no evidence of heterogeneity was observed for associations between dietary or circulating beta-carotene and all-cause mortality. However, some limitations of our meta-analysis should be considered. First, observational studies were susceptible to undiscovered confounders, such as unadjusted lifestyle, specific nutrients, *et al.* Second, the number of studies was limited to draw a firm conclusion. However, on basis of current evidence, beta-carotene from natural food may seem favorable to our body. Third, we did not conduct dose-response analysis for lack of available data even we had tried our best to connect the authors to obtain essential information. Further study should be conducted to explore dose-response relation in general population hereafter.

In summary, the current meta-analysis shows that both dietary intake and circulating level of beta-carotene were inversely associated with the risk of all-cause mortality. More studies should be conducted in various populations with different diet habits to clarify the dose-response relation in order to determine optimal intake for dietary guidance in terms of public health policy and practice.

## Methods

### Data sources, search strategy, and selection criteria

We followed standard criteria for conducting and reporting of the current meta-analyses^44^. We conducted a comprehensive publication search in the database of PubMed and EMBASE up to 31 March 2016 for studies assessing the relationship of dietary intake or/and blood concentrations of beta-carotene with all-cause mortality risk. The search terms used were as following: (mortality OR death) AND (antioxidant OR carotenoid OR carotene) AND (cohort studies OR follow-up studies OR longitudinal studies OR prospective studies).

The publication can be included only if it: (1) was a prospective cohort, case-cohort, or nested case-control study conducted in general healthy population, (2) reported dietary intake of beta-carotene or blood beta-carotene concentration as exposure status, (3) presented total mortality as the outcome of interest, (4) provided information about relative risk (RR) and the corresponding 95% confidence interval (CI) or data necessary to calculate these. In addition, we manually reviewed the references list of all previous reviews, relevant meta-analysis and the studies that were included in this analysis to identify other eligible articles that were not found in preliminary document retrieval. When the studies with same population were published repeatedly, priority was given to the publication with largest number of cases or which with most applicable estimates.

### Data collection

Two investigators (Long-Gang Zhao and Qing-Li Zhang) independently reviewed all available studies and extracted data with a standard collection form. Any discrepancies were discussed and resolved by the two authors. The following characteristics in the identified studies were recorded: first author’s last name, year of publication, country in which the study was conducted, study name, study period, response rate, follow-up rate, sample size, total number of death, population age at baseline, method used to assess dietary intake of beta-carotene, median or mean dietary intake or circulating level of beta-carotene, categories of dietary intake of beta-carotene or circulating concentration and the RRs or hazards ratio (HRs) and 95%CIs for all-cause mortality associated with those categories, gender of study population, and covariates included for adjustment in multivariable regression models. If multiple estimates were provided, results with multivariable-adjusted risk estimates that were adjusted for most potential confounding factors in original studies were adopted.

### Statistical analysis

For two studies reported the RR for per standard deviation^10^,^26^, we translated it into RR for top tertile versus bottom tertile using method introduced by Danesh *et al.*^45^. For the studies^17^,^19^,^22^ reported sex-specific results only, we first combined the separate data in a fixed model and then incorporated them with other studies.

In order to examine the association between beta-carotene intake, circulating beta-carotene concentration and risk of all-cause mortality, we calculated pooled RR and 95% CI comparing the highest with the lowest category of beta-carotene intake or blood concentration. We adopted the random-effect model to account for variation between studies proposed by DerSimonian and Laird^46^. The possible heterogeneity among studies was tested using the Q (*χ*^*2*^) and *I*^*2*^ statistics. Meta-regression was performed to investigate potential sources of heterogeneity between studies. Critical heterogeneity was defined when *I*^*2*^ > 50% or *P*_for Q statistic_ < 0.10^47^.

We did not assess study quality but we conducted stratified analysis by such specific study characteristics as duration of follow-up, cohort size, exposure measurement types, population age at baseline, median exposure level and adjustment for confounders (history of chronic disease, smoking status and alcohol consumption, body mass index [BMI], physical activity, vitamin supplements use, serum cholesterol level), which are indicators of study quality. Furthermore, we performed sensitivity analyses by excluding one study at a time to test the influence of individual study on the pooled estimate. Potential publication bias was evaluated by visual inspection of funnel plot and formal testing using Egger’s and Begg’s tests^48^.

All statistical analyses were carried out with Stata (version 13.0). *P* values were two-sided with a significance level of 0.05 if not specified.

## Additional Information

**How to cite this article**: Zhao, L.-G. *et al.* Dietary, circulating beta-carotene and risk of all-cause mortality: a meta-analysis from prospective studies. *Sci. Rep.* **6**, 26983; doi: 10.1038/srep26983 (2016).

## Supplementary Material

Supplemental Figure 1

## Figures and Tables

**Table 1:** General characteristics of prospective studies of dietary or serum beta-carotene and all-cause mortality (1997–2016).

No.	First author, year	Country	Cohort or Location	Response rate	Follow-up years	Follow-up rate	Cohort size	No. of death	Baseline age (year)	Exposure measurement	Median	Quantity	Sex	Adjustment
1	Stepaniak, 2016	Eastern Europe	HAPIEE study	59.00%	7.2	95.60%	26993	2371	45–69	Validated FFQ	7404.7 ug/d	Quintile 5 vs. Q1; 13955.3/3189.5	Both	Age, country, education, smoking status, alcohol intake, body mass index, hypertension, diabetes, hypercholesterolemia, history of cadiovascular disease or cancer, total energy intake
2	Henríquez-Sánchez, 2016	Spain	PREDIMED study	Not available	4.3	97.20%	7015	319	M: 55–80 F: 60–80	Validated FFQ	Not available	Quintile 5 vs. Q1	Both	Recruitment center, intervention group, age, sex, education, marital status,body mass index, smoking habit, alcohol consumption, total energy intake, energy-adjusted intake of saturated fatty acids, polyunsaturated fatty acids, monounsaturated fatty acids and glycemic index and medical history of hypertension, diabetes, dyslipidemia and cancer.
3	Roswall, 2012	Danmark	DCH study	35.50%	13.8	100.00%	55453	6767	50–64	Validated FFQ	3205.4 ug/d	Highest vs. Lowest group; >4798/<1317	Both	Age, alcohol intake, body mass index, waist circumference, smoking status, smoking duration, smoking intensity, time since cessation, education, and physical activity, vitamin E, vitamin C, folic acid, vitamin supplementation.
4	Agudo, 2008	Spain	EPIC-Spain	55%-60%	6.5	100.00%	41358	562	30–69	Validated dietary histogoty	1678.6 ug/d	Quartile 4 vs. Q1; 3707.2/830.4	Both	Age, sex, total energy intake, education, body mass index, physical activity, cigarette smoking, and alcohol consumption.
5	Genkinger, 2004	US	CLUE cohort studies	86.00%	12.2	97.00%	6151	910	30–93	FFQ	1697.0 ug/d	Quintile 5 vs. Q1; 3884.8/679.2	Both	Age, smoking status, body mass index, cholesterol concentration, and energy.
6	Fletcher, 2003	UK	Substudy of a randomized trial	47%(Dietary); 52%(Plasma)	4.4	100.00%	1175	290	75–84	FFQ	2154 ug/d	Quintile 5 vs. Q1;	Both	Age, sex, total energy intake, body mass index, cholesterol, systolic blood pressure, smoking, alcohol, diabetes, history of cardiovascular disease or cancer, supplement use, physical activity, and housing tenure
										Plasma	372 nmol/L	Quintile 5 vs. Q1; 772/153	Both	Age, sex, body mass index, cholesterol, systolic blood pressure, smoking, alcohol, diabetes, and history of cardiovascular disease or cancer, physical activity, housing tenure, vitamin supplementation.
7	Todd, 1999	UK	SHHS	Not available	7.7	99.90%	11629	591	40–59	FFQ	2967.7 ug/d	Quartile 4 vs. Q1	Both	Age, serum total cholesterol, systolic btood pressure, carbon monoxide, energy, previous medical diagnosis of diabetes, body mass index, the Bortner personality score, trigtycerides, high density llpoproteln cholesterol, fSrinogen, a self-reported measure of activity in leisure, and alcohol consumption
8	Goyal, 2013	US	NHANES III	78%	14.2	96.70%	16008	4225	>20	Serum	368 nmol/L	Quintile 5 vs. Q1; >520/<130	Both	Age, sex, race-ethnicity, level of education, annual family income, body mass index, smoking status, serum cotinine level, alcohol consumption, fruit and vegetable intake, physical activity, serum total cholesterol levels, hypertension status, diabetes status, history of heart attack, congestive heart failure, stroke or cancer, hormone use in women, and supplement use
9	Bates, 2011	UK	BNDNs	99.80%	13.5	94.50%	1054	717	>65	Plasma	363 nmol/L	per SD	Both	Age and sex
10	Huerta, 2006	Spain	Asturias	Not available	4.3	96.00%	154	31	61.5–79.8	Serum	168 nmol/L	Tertile 3 vs. T1; >177.51/<87	Both	Age, sex, body mass index, self-perceived health, alcohol consumption, practice of daily exercise, diabetes, use of antihypertensive drugs, plasma albumin concentration, plasma lipids.
11	Ito, 2002	Japan	CHEP (1990–1994)	Not available	6–10	90.50%	2444	146	39–80	Serum	455 nmol/L	Tertile 3 vs. T1	Both	Age, sex, habits of smoking and alcohol consumption, and serum levels of total cholesterol and GPT activity
12	Kilander, 2001	Sweden	Uppsala	82.00%	22.7–25.7	100.00%	2285	630	48.6–51.1	Serum	302 nmol/L	per SD	Male	Age
13	Ito, 1997	Japan	CHEP (1986–1989)	Not available	2–8	Not available	2348	98	39–83	Serum	666 nmol/L	Highest vs. Lowest group; Male, >592/266; Female, 1266/682	Both	Age, sex, smoking, alcohol drinking.

Abbreviation: FFQ, food frequency questionaire; SD, standard deviation; M, male; F, female.

**Table 2:** Stratified pooled relative risks and 95% confidence intervals for highest versus lowest category of dietary beta-carotene and all-cause mortality.

Subgroups		N	Death	Participants	RR	95%CI	*I*^*2*^ (%)	*P*_*a*_	*P*_*b*_
All studies		7	11810	149774	0.83	0.78, 0.88	1.00	0.416	
Duration of follow-up									0.144
	<10 years	5	4133	88170	0.89	0.80, 0.98	0	0.555	
	> = 10 years	2	7677	61604	0.79	0.73, 0.86	0	0.826	
Sample size									0.902
	<10,000	3	1519	14341	0.83	0.70, 0.97	0	0.928	
	> = 10,000	4	10291	135433	0.84	0.75, 0.94	49.30	0.116	
Population age at baseline									0.255
	<50	4	4434	86131	0.87	0.78, 0.97	14.50	0.320	
	> = 50	3	7376	63643	0.80	0.74, 0.86	0	0.843	
Median intake									0.519
	<2500 ug/d	3	1762	48684	0.80	0.69, 0.92	0	0.722	
	> = 2500 ug/d	3	9729	94075	0.86	0.75, 0.99	60.80	0.078	
Validated FFQ									0.751
	Yes	4	10019	130819	0.83	0.74, 0.93	42.70	0.155	
	No	3	1791	18955	0.85	0.73, 1.00	0	0.741	
Major confounders adjusted									
History of disease									0.071
	Yes	4	3571	46812	0.79	0.73, 0.85	0	0.852	
	No	3	8239	102962	0.92	0.82, 1.03	0	0.920	
Smoking and drinking									0.837
	Yes	5	10309	131994	0.83	0.76, 0.91	25.60	0.251	
	No	2	1501	17780	0.85	0.72, 1.00	0	0.451	
Physical activity									0.158
	Yes	4	8210	109615	0.80	0.74, 0.86	0	0.629	
	No	3	3600	40159	0.89	0.80, 1.00	0	0.457	
Serum cholesterol level									0.751
	Yes	3	1791	18955	0.85	0.73, 1.00	0	0.741	
	No	4	10019	130819	0.83	0.74, 0.93	42.70	0.155	
Vitamin supplementation use									0.302
	Yes	2	7057	56628	0.79	0.73, 0.86	0	0.574	
	No	5	4753	93146	0.87	0.79, 0.96	0	0.461	

Abbreviation: RR, relative risk; CI, confidence interval; *I* ^*2*^, measure of heterogeneity; *P*_*a*_, P value for heterogeneity within each group; *P*_*b*_, P value for heterogeneity between subgroups in meta-regression.

**Table 3:** Stratified pooled relative risks and 95% confidence intervals for highest versus lowest category of circulating beta-carotene and all-cause mortality.

Subgroups		n	Death	Participants	RR	95%CI	*I*^*2*^ (%)	*P*_*a*_	*P*_*b*_
All studies		7	6137	25468	0.69	0.59, 0.80	37.10	0.145	
Duration of follow-up									0.496
	< 10 years	4	565	6121	0.62	0.47, 0.82	0	0.494	
	> = 10 years	3	5572	19347	0.71	0.57, 0.88	67.20	0.047	
Sample size									0.246
	<2,000	3	1038	2383	0.81	0.67, 0.97	0	0.600	
	> = 2,000	4	5099	23085	0.63	0.51, 0.78	46.60	0.132	
Population age at baseline									0.246
	<50	4	5099	23085	0.63	0.51, 0.78	46.60	0.132	
	> = 50	3	1038	2383	0.81	0.67, 0.97	0	0.600	
Median level									0.076
	<350 nmol/L	2	661	2439	0.54	0.42, 0.70	0	0.688	
	> = 350 nmol/L	5	5476	23029	0.75	0.66, 0.86	9.30	0.354	
Blood sample									0.197
	Serum	5	5130	23239	0.63	0.52, 0.77	34.00	0.194	
	Plasma	2	1007	2229	0.82	0.68, 0.99	0	0.741	
Major confounders adjusted									
History of disease									0.560
	Yes	3	4546	17337	0.74	0.63, 0.88	0	0.703	
	No	4	1591	8131	0.64	0.49, 0.84	63.80	0.040	
Smoking and drinking									0.962
	Yes	4	4759	21975	0.71	0.61, 0.83	1.40	0.385	
	No	3	1378	3493	0.66	0.46, 0.96	69.20	0.039	
BMI									0.560
	Yes	3	4546	17337	0.74	0.63, 0.88	0	0.703	
	No	4	1591	8131	0.64	0.49, 0.84	63.80	0.040	
Physical activity									0.572
	Yes	4	4692	19781	0.74	0.63, 0.86	0	0.839	
	No	3	1445	5687	0.62	0.44, 0.89	75.90	0.016	
Serum cholesterol level									0.471
	Yes	3	4661	19627	0.74	0.63, 0.87	0	0.918	
	No	4	1476	5841	0.61	0.44, 0.85	65.90	0.032	
Vitamin supplementation use									0.454
	Yes	2	4515	17183	0.75	0.64, 0.89	0	0.960	
	No	5	1622	8285	0.63	0.49, 0.82	54.60	0.066	

Abbreviation: RR, relative risk; CI, confidence interval; *I* ^*2*^, measure of heterogeneity; *P*_*a*_, P value for heterogeneity within each group; *P*_*b*_, P value for heterogeneity between subgroups in meta-regression.
