# Supplementary material for: Dietary, circulating beta-carotene and risk of all-cause mortality: a meta-analysis from prospective studies
Source: Sci Rep. 2016 May 31;6:26983. doi: 10.1038/srep26983 (PMC4886629; doi:10.1038/srep26983)
Supplement: Supplemental Figure 1 [file srep26983-s1.pdf]

Dietary, circulating beta-carotene and risk of all-cause mortality: a meta-analysis from prospective studies

Long-Gang Zhao, Qing-Li Zhang, Jia-Li Zheng, Hong-Lan Li, Wei Zhang, Wei-Guo Tang, Yong-Bing Xiang

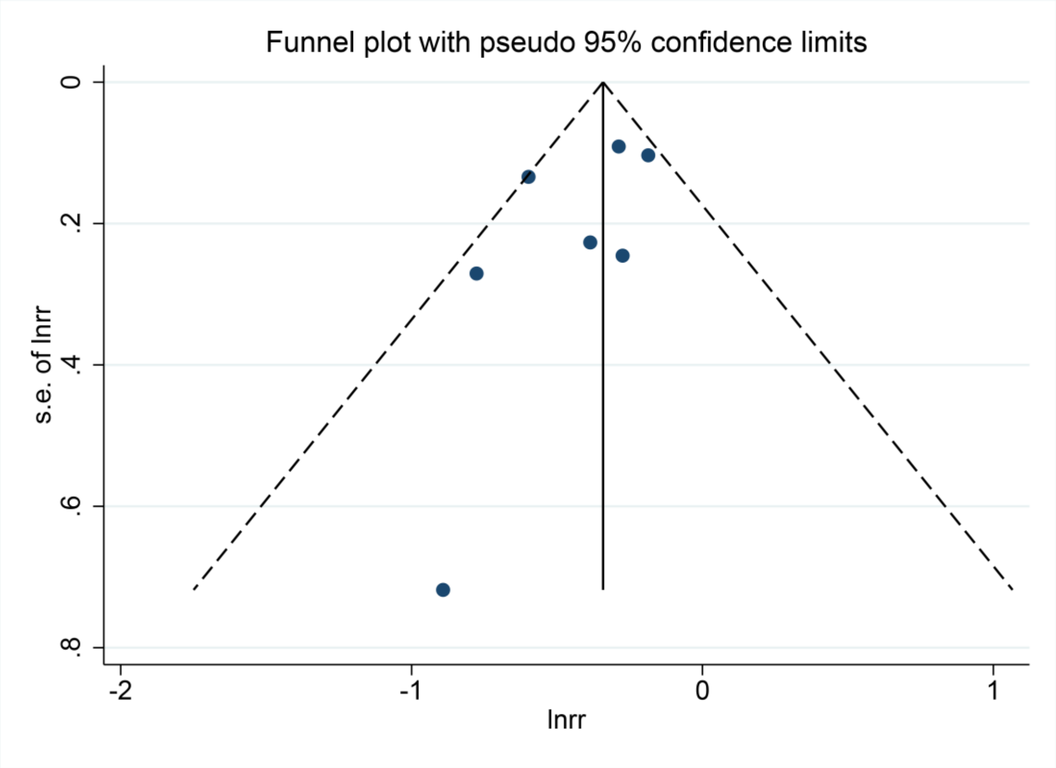

Supplemental Figure 1. Funnel plot for studies of the association between dietary intake of beta-carotene and all-cause mortality.

Each dot indicates one study with its size and its relative risk (RR). The y-axis contains the SEs of the ln (RR). The smaller the SE, the larger the study. The ln (RR) are plotted on the x-axis. The lines are drawn around the dots to visualize how symmetrically the studies are divided around the true estimate; symmetry indicates no evidence of publication bias.

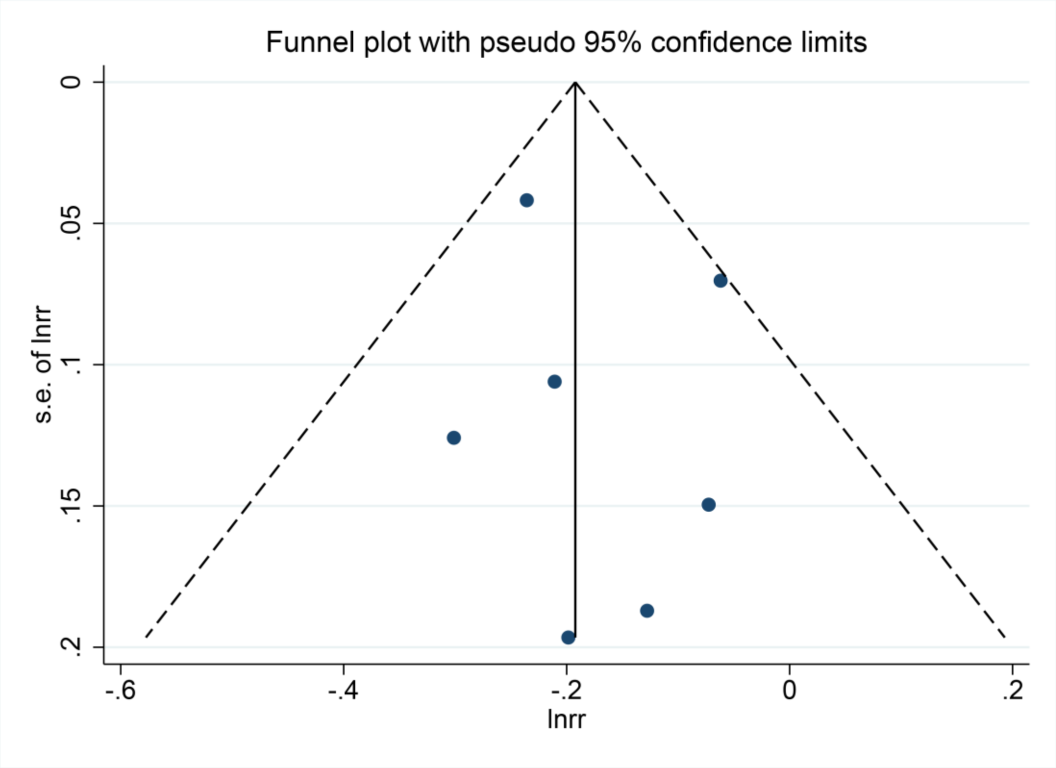

Supplemental Figure 2. Funnel plot for studies of the association between circulating level of beta-carotene and all-cause mortality.

Each dot indicates one study with its size and its relative risk (RR). The y-axis contains the SEs of the ln (RR). The smaller the SE, the larger the study. The ln (RR) are plotted on the x-axis. The lines are drawn around the dots to visualize how symmetrically the studies are divided around the true estimate; symmetry indicates no evidence of publication bias.
